# Precise diagnosis and risk stratification of prostate cancer by comprehensive serum metabolic fingerprints: a prediction model study

**DOI:** 10.1097/JS9.0000000000001033

**Published:** 2024-01-04

**Authors:** Xiaochen Fei, Xinxing Du, Jiayi Wang, Jiazhou Liu, Yiming Gong, Zejun Zhao, Zhibin Cao, Qibo Fu, Yinjie Zhu, Liang Dong, Baijun Dong, Jiahua Pan, Wenshe Sun, Shaowei Xie, Wei Xue

**Affiliations:** a Department of Urology; b Department of Ultrasound, Ren Ji Hospital, Shanghai Jiao Tong University School of Medicine; c Department of Urology, Jiading District Central Hospital, Shanghai University of Medicine and Health Sciences, Shanghai; d Medical Science and Technology Innovation Center, Shandong First Medical University and Shandong Academy of Medical Sciences, Shandong, People’s Republic of China

**Keywords:** mass spectrometry, matrix-assisted laser desorption ionization, metabolomic, prostate neoplasms

## Abstract

**Objectives:**

Prostate cancer (PCa) is one of the most common malignancies in men worldwide and has caused increasing clinical morbidity and mortality, making timely diagnosis and accurate staging crucial. The authors introduced a novel approach based on mass spectrometry for precise diagnosis and stratification of PCa to facilitate clinical decision-making.

**Methods:**

Matrix-assisted laser desorption ionization time-of-flight (MALDI-TOF) mass spectrometry analysis of trace blood samples was combined with machine learning algorithms to construct diagnostic and stratification models. A total of 367 subjects, comprising 181 with PCa and 186 with non-PCa were enrolled. Additional 60 subjects, comprising 30 with PCa and 30 with non-PCa were enrolled as an external cohort for validation. Subsequent metabolomic analysis was carried out using Autoflex MALDI-TOF, and the mass spectra were introduced into various algorithms to construct different models.

**Results:**

Serum metabolic fingerprints were successfully obtained from 181 patients with PCa and 186 patients with non-PCa. The diagnostic model based on the eight signals demonstrated a remarkable area under curve of 100% and was validated in the external cohort with the area under curve of 87.3%. Fifteen signals were selected for enrichment analysis, revealing the potential metabolic pathways that facilitate tumorigenesis. Furthermore, the stage prediction model with an overall accuracy of 85.9% precisely classified subjects with localized disease and those with metastasis. The risk stratification model, with an overall accuracy of 89.6%, precisely classified the subjects as low-risk and high-risk.

**Conclusions:**

Our study facilitated the timely diagnosis and risk stratification of PCa and provided new insights into the underlying mechanisms of metabolic alterations in PCa.

## Background

Highlights

- Matrix-assisted laser desorption ionization time-of-flight mass spectrometry serves as a promising and reliable technique that helps to accomplish a comprehensive metabolic analysis of diverse malignancies.
- Serum metabolic fingerprints analysis is promising for universal and large-scale point-of-care applications.
- Diagnostic models based on serum metabolic fingerprints have exhibited great potential in improving clinical staging and risk stratification in a timely, cost-effective, and efficient manner.

Prostate cancer (PCa) is one of the most common malignancies with 1 414 259 new cases each year in men globally, leading to substantial clinical mortality with ~375 304 deaths annually^1,2^. The timely identification and proper management of PCa are crucial for enhancing long-term clinical outcomes^3–5^. Despite the widespread use of serum prostate-specific antigen (PSA) for PCa-screening in clinical practice, its diagnostic accuracy may be compromised by elevated levels in certain benign conditions, resulting in suboptimal sensitivity and specificity^6,7^. Meanwhile, MRI with a high negative predictive value, can obviate unnecessary biopsy and improve the overall diagnosis^8^. Nonetheless, it is inevitable to misdiagnose some clinically significant cancers due to the multifocal nature of disease within the prostate, let alone the high costs, experienced radiologist dependency, and time consumption^9^. Hence, there is still a critical and unmet need to identify specific biomarkers for time detection and risk stratification of PCa. Liquid biopsy, which detects tumor-related biomarkers from body fluids in a noninvasive and comprehensive manner, represents a reliable tool for cancer diagnosis^10^. In this context, new strategies to expedite accurate and precise diagnosis based on a substantial number of liquid biomarkers, including targeting proteomics and transcriptomics, circulating tumor cells, and extracellular vesicles, are actively being sought^11–13^.

Metabolic reprogramming is recognized as an important hallmark of malignancy, rendering it a promising target for cancer diagnosis, monitoring, and treatment^14–16^. Accumulating evidence implicates that metabolic abnormalities fuel the growth and proliferation of tumor cells^17–19^. Given that metabolites are the ultimate biochemical products of gene regulation and protein function, metabolomic profiling is able to rapidly and accurately reveal the biological state of living systems^20^. Indeed, prior studies on PCa have underlined the potential significance of metabolomics in disease diagnosis and evaluation^21–23^. The past decade has witnessed significant development in the methodologies for metabolite analysis, including nuclear magnetic resonance spectroscopy and mass spectrometry (MS)^24–27^. Long used in analytical chemistry, MS is capable of discovering and identifying distinct cancer-specific metabolites, but its analytical performance is highly hindered by complex sample pretreatment, low sensitivity in the detection of macromolecules, and high cost^28,29^. On the contrary, matrix-assisted laser desorption ionization time-of-flight (MALDI-TOF) MS, as a new nanoparticle-based MS technology, has demonstrated the ability to effectively identify a diverse range of metabolites within various bodily fluids in a rapid, accurate, and economically efficient manner^30–34^. Therefore, MALDI-TOF MS serves as a promising and reliable technique that helps to accomplish a comprehensive metabolic analysis of PCa.

**Figure 1:**
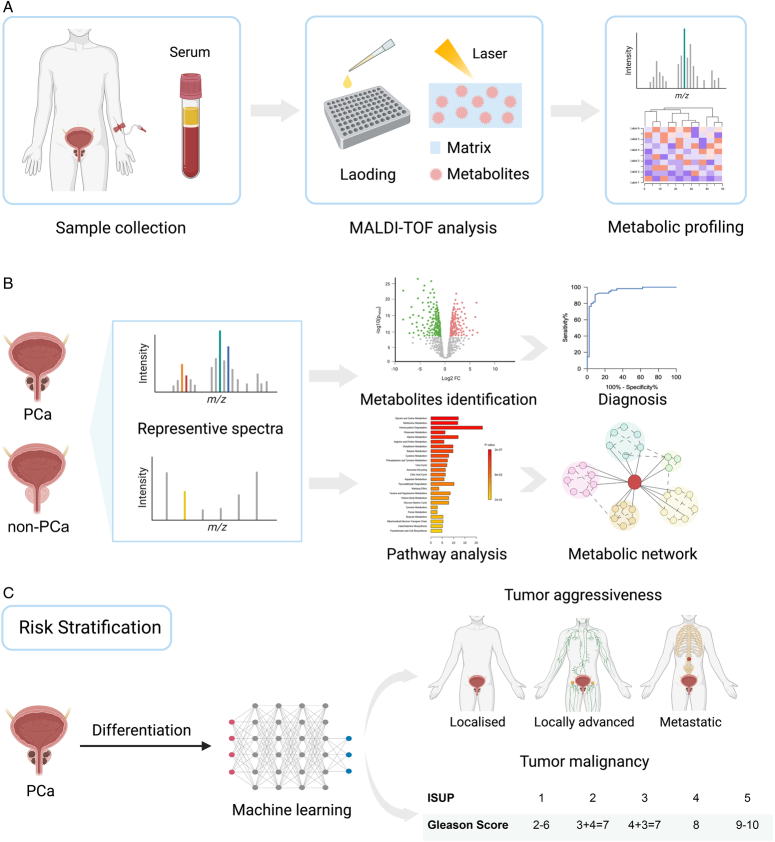
The workflow of metabolic profiling based on serum samples for precise diagnosis and risk stratification of prostate cancer (PCa). (A) Serum samples from the enrolled subjects were loaded and tested by matrix-assisted laser desorption ionization time-of-flight mass spectrometry. (B) Serum metabolic fingerprints were analyzed by different algorithms to distinguish the PCa subjects from the non-PCa subjects, and the metabolic pathway and network analysis were conducted. (C) Machine learning of serum metabolic fingerprints from the PCa subjects included feature selection and model building to predict the risk group and clinical stage.

Thus, we performed comprehensive metabolomic profiling of the blood samples from 367 enrolled subjects (181 subjects with PCa and 186 subjects with non-PCa) using MALDI-TOF MS. In this study, we acquired serum metabolic fingerprints (SMFs) with high sensitivity, reproducibility, and stability in a fast, high-throughput, and cost-effective manner (Fig. 1A). Second, a diagnostic model with high-performance was developed using fingerprint spectra, demonstrating a remarkable accuracy of 100%, which was validated in an external cohort of 60 subjects with an accuracy of 87.3%. Moreover, potential metabolic pathways that facilitate tumorigenesis were identified (Fig. 1B). Furthermore, we built a risk and stage stratification model by machine learning that effectively screened PCa patients at high-risk (Fig. 1C). Accordingly, our work facilitates the timely diagnosis and disease stratification of PCa and provides new insights into the underlying mechanisms of metabolic alterations in PCa.

## Methods

### Enrolled population and eligibility criteria

Of the screened population with elevated PSA values visiting Ren Ji Hospital (Shanghai, China) from October 2021 to March 2022, 387 subjects who met all the following criteria were included in the present study: (1) they had a serum PSA value higher than 4 ng/ml or abnormal ultrasound findings, including focal hypoechoic in peripheral zone, or focal contour bulge or definite extra-prostatic extension/invasive behavior, or asymmetric/increased blood flow in prostate; (2) they underwent 12-core transrectal ultrasound-guided prostate biopsy; (3) they underwent pelvic MRI to evaluate the primary lesion and bone scan or PET/CT to assess the distant metastases; (4) they underwent a fasting venous blood draw before the biopsy procedure. Subjects with other types of malignancies, systemic diseases, comorbidities, and metabolic syndrome were excluded from the present study. In addition, a total of 60 subjects from Jiading District Central Hospital affiliated with Shanghai University of Medicine and Health Science were also enrolled as an external cohort for model validation. This study was conducted in accordance with the principles of the Declaration of Helsinki, and was approved by the Ethics Committee of Ren Ji Hospital, Shanghai Jiao Tong University School of Medicine (KY2021-030). The signed informed consents were obtained from all participants or their authorized representatives prior to serum sample collection. All subjects were diagnosed by a panel of urologists and pathologists according to the biopsy tissue pathology results. Disease staging and pathological grading of PCa were defined according to the European Association of Urology (EAU) guidelines and the International Society of Urological Pathology (ISUP) 2014 grade system^35,36^. The present work has been reported in line with the TRIPOD (transparent reporting of a multivariable prediction model for individual prognosis or diagnosis) guidelines^37^ (Supplemental Table S1, Supplemental Digital Content 1, http://links.lww.com/JS9/B621).

### Serum sample collection

After at least 8 h of overnight fasting, the skin of the subjects was disinfected, and 5 ml of peripheral venous blood was collected from the subjects and stored with a coagulant tube at room temperature for stratification. The blood samples collected were handled carefully, and the tubes were closely plugged and placed vertically to avoid cross-contamination. Then, the blood was centrifuged at 3000×g for 10 min, and 500 μl of supernatant (i.e. serum) was collected after the coagulant tube was placed for 30 min. The collected serum was immediately preserved in the Eppendorf (EP) tube at −80°C until analysis. The serum should not be freeze-thawed repeatedly. To make sure that the serum samples were identical with the subjects, the tubes were labeled by names and the hospitalization numbers. To prevent the potential biases due to sample collection, all serum samples collected at different times were stored in the randomized order. The researchers were blind to the sample classification during the sample analysis. Before MALDI-TOF MS analysis, all samples were thawed in an ice box with crushed ice at a temperature range of 2–4°C and homogenized by a vortex mixer before loading to the plate.

### Matrix construction

To synthesize molybdenum disulfide (MoS_2_) compounds, 10 mg Molybdenum oxide was firstly added to 1 ml of a purified water solution containing 12 mg PVP. The mixture was magnetically stirred for 15 min and 25 mg sodium sulfocyanate was substantially added with vigorous stirring for 20 min. Then, the mixture was heated at 200°C for 36 h in a Teflon stainless autoclave. The obtained product was centrifuged and cleaned three times with deionized water and ethanol, respectively. Lastly, the MoS_2_ nanoparticles were dried at 75°C until further use. To synthesize two-dimensional carbon-nitrogen compound C_3_N_4_, melamine, and dicyandiamide were mixed at the mass ratio of 4.0:3.2 and heated at 500°C for 4 h. After cooling down to room temperature, the mixture was centrifuged and cleaned three times with deionized water and ethanol to obtain the final product C_3_N_4_. Subsequently, the MoS_2_ was mixed into C_3_N_4_ at a mass ratio of 1:10–20 and thermally polymerized at 140–160°C for 4–5 h to obtain a MoS_2_/C_3_N_4_ heterojunction composite matrix.

### Metabolomics analysis

An Autoflex MALDI-TOF (Bruker Daltonics, Germany) equipped with a Nd:YAG laser (wavelength of 355 nm) was utilized to perform metabolomic analysis of targeted samples (i.e. serum) and standard metabolites. The process involved spotting 0.3 μl samples on an MTP 384 polished steel plate, air-drying them with 0.5 μl MoS2/C3N4 matrices, and acquiring MS spectra in reflection mode with a laser frequency of 1500 Hz. The acquired spectra were automatically analyzed using FlexAnalysis software. To confirm the validity of the aforementioned analysis, a standard sample consisting of creatinine, arginine, and methionine (1 ng/l) was tested. Additionally, 0.5 M NaCl and 5 mg/ml bovine serum albumin (BSA) were introduced to the standard leucine sample (1 ng/nl) to evaluate its tolerance to salt and protein.

### Statistical analysis

Metabolites were identified based on their mass charge ratio (m/z) using the Human Metabolome Database (https://hmdb.ca/spectra/ms/search) and MetFrag (https://ipb-halle.github.io/MetFrag). Initially, unsupervised principal component analysis (PCA) was performed to transform the m/z signals into linearly uncorrelated variables in order to lower the dimensionality of the data and identify the natural clustering. The first principal component accounted for the largest variance. The PCA score plot was then visualized by placing the top two principal components in a two-dimensional matrix. Subsequently, a supervised orthogonal partial least squared discriminant analysis (OPLS-DA) was performed to maximize detection of m/z signals associated with differentiation between the known classes for disease prediction. As criterion to assess the performance of the models, R2Y (goodness of fit parameter) and Q2 (predictive ability parameter) >0.5 for OPLS-DA were adopted^38,39^. Both PCA and OPLS-DA were performed using MetaboAnalyst 5.0 (https://www.metaboanalyst.ca/home.xhtml). Volcano curve, heatmap, enrichment analysis, and metabolic pathway analysis were also performed using the above software. For volcano plots, each m/z feature with a fold change (FC) of >2 and *t*-test threshold *P*-value <0.05 was set as the parameter. The receiver operative curve was used for the model evaluations and comparisons. Area under curve (AUC), sensitivity, and specificity were calculated for the quantitative comparisons. Machine learning of the SMFs, including feature selection and model building, was performed for disease stratification models. For feature selection, variance filtering was used to select the candidate m/z features, and univariate feature selection methods, including the χ^2^test, analysis of variance F-value (ANOVA F), and mutual information (MI), were subsequently performed. For model building, random forest (RF) was applied to obtain the optimized model using fivefold cross-validation. The classification performance of the disease stratification models was evaluated by accuracy, F1 score, and precision. The output models were, respectively, tested in the validation cohort and the external cohort to evaluate their performance.

## Results

### High-performance of MALDI-TOF analysis

**Figure 2:**
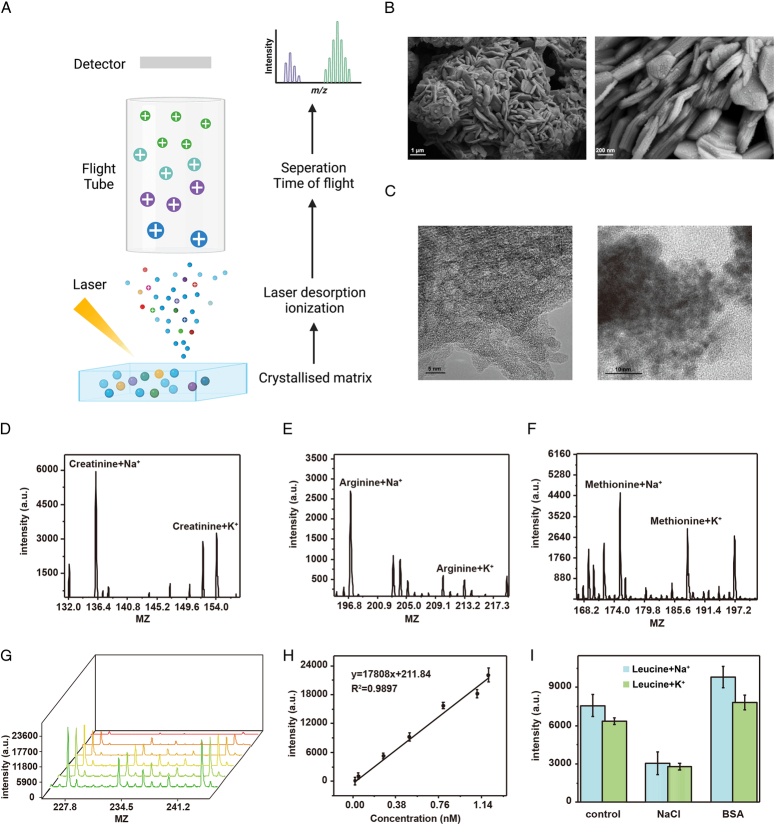
Characterization and high-performances of the matrix. (A) The workflow of matrix-assisted laser desorption ionization time-of-flight mass spectrometry analysis. (B) Scanning electron microscopy images of MoS_2_/C_3_N_4_. (C) Transmission electron microscopy images of MoS_2_/C_3_N_4_. (D-F) Performances of MoS_2_/C_3_N_4_ for the detection of creatinine, arginine, and methionine (1 ng/nl). (G) The mass spectrometry spectra of MoS_2_/C_3_N_4_ for detecting leucine of different concentrations (0.05–0.8 mM) (H) The corresponding calibration curve of MoS_2_/C_3_N_4_ for detecting leucine. (I) Salt tolerance and protein tolerance of MoS_2_/C_3_N_4_ for quantification of 1 ng/nl leucine in 0.5 M NaCl and 5 mg/ml bovine serum albumin.

We successfully constructed MoS_2_/C_3_N_4_ nanoparticles as matrices for subsequent MS analysis (Fig. 2A). The morphology of the nanoparticles was examined using scanning electron microscopy and transmission electron microscopy (TEM), which revealed thin sheet-like structures and a uniform rough surface (Fig. 2B and C). Other supporting characterizations of the MoS_2_/C_3_N_4_ nanoparticles are summarized in the Supplementary Information (Supplemental Digital Content 2, http://links.lww.com/JS9/B622), including elemental mapping analysis (Figure. S1a-d, Supplemental Digital Content 2, http://links.lww.com/JS9/B622), and high-resolution TEM (HRTEM) (Figure. S1e, Supplemental Digital Content 2, http://links.lww.com/JS9/B622). Next, we confirmed the good performance of MoS_2_/C_3_N_4_ for the detection of standard small metabolites (1 ng/l), including creatinine, arginine, and methionine (Fig. 2D–F). In addition, the matrix displayed distinct linearity for leucine quantification (y=17808x + 211.84, R²=0.9897), ranging from 0.05 to 0.8 mM (Fig. 2G, H). Notably, the matrix also achieved high stability in the detection of metabolite mixtures containing high concentrations of NaCl (0.5 M) and BSA (5 mg/ml), indicating its applicability in analyzing intricate biological samples (Fig. 2I).

### Characterization of PCa-specific SMFs for clinical diagnosis

**Figure 3:**
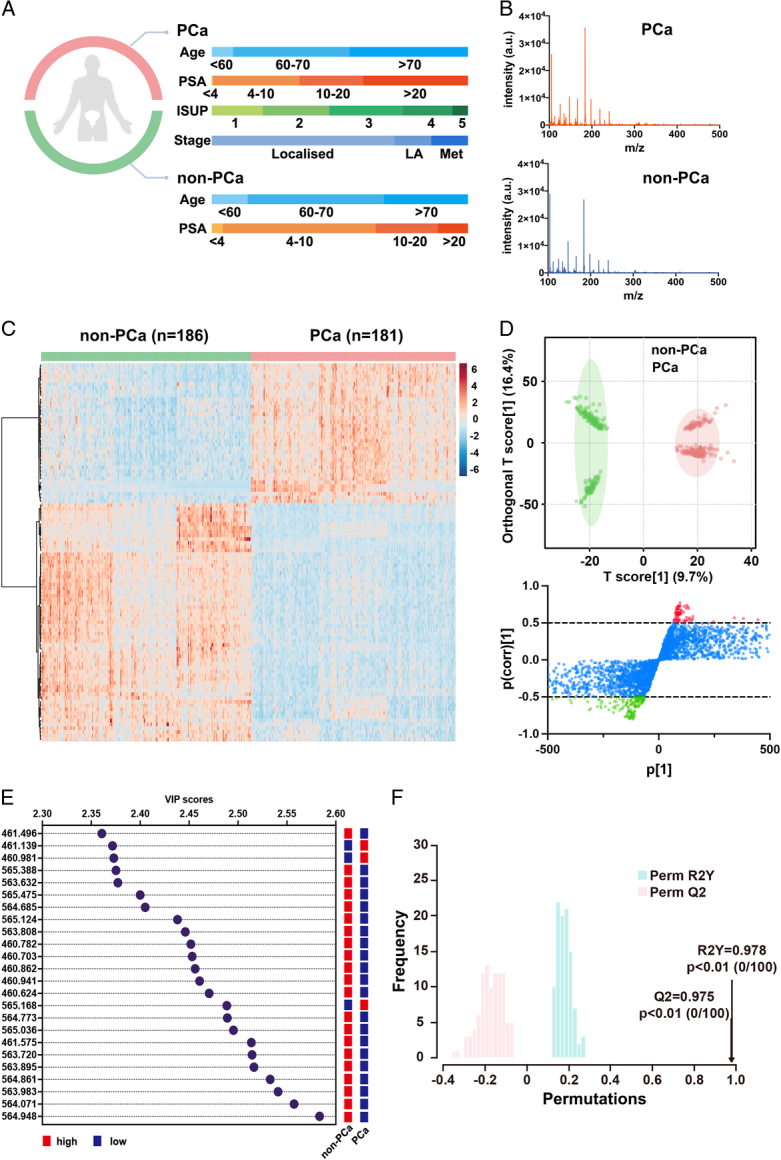
Characterization and classification of serum metabolic fingerprints for prostate cancer (PCa) diagnosis. (A) The clinical features of 181 subjects with PCa and 186 subjects with non-PCa. (B) The representative mass spectrometry spectra of the serum samples of PCa and non-PCa subjects at the m/z range of 100–500. (C) The heatmap of serum metabolic fingerprints matrix was plotted using 100 m/z signals. (D) The orthogonal partial least squared discriminant analysis (OPLS-DA) classification for the PCa (red points) and non-PCa (green points) subjects, and S-plot with the most upregulated (red points) and downregulated (green points) m/z signals. (E) The parameters of variable importance in the projection for the OPLS-DA model. (F) The permutation test of the OPLS-DA model.

Direct mass spectra were obtained from serum samples of 367 subjects, including 181 subjects with PCa and 186 subjects with non-PCa. The clinical features of the patients are shown in Figure 3A, and Supplementary Table S2 (Supplemental Digital Content 3, http://links.lww.com/JS9/B623). The m/z signals from 100 to 600 Da for each sample were generated, depicting the small metabolites in the low mass range. Figure 3B shows one of the typical mass spectra results of serum samples from a subject with PCa and a subject with non-PCa. Next, we extracted the m/z signals from the raw data as the SMFs of each serum sample, which demonstrated significant differences between the two cohorts (Fig. 3C).

To further distinguish the serum samples, a conventional PCA algorithm was performed based on SMFs, which demonstrated that the serum samples from PCa and non-PCa subjects appeared to be roughly divided into two clusters, as shown in Figure S2 (Supplemental Digital Content 2, http://links.lww.com/JS9/B622). Subsequently, we implemented the OPLS-DA algorithm to achieve distinct classification of the two cohorts. Figure 3D shows the m/z signals with significant differences between subjects with PCa and those with non-PCa. Notably, 88 m/z signals marked in red were upregulated, and 164 m/z signals marked in green were downregulated in subjects with PCa. To evaluate the impact of different m/z signals on the OPLS-DA model, variable importance in the projection (VIP) scores were calculated, as shown in Figure 3E, where m/z 564.948, 564.071, 563.983, 564.861, and 563.895 made enormous contributions to the OPLS-DA model. To further evaluate the performance of the model, a permutation test was conducted with 100 iterations, which demonstrated that the model achieved an optimal level of Q2=0.975 and R2Y=0.978.

### Identification of PCa-specific metabolic biomarkers for clinical diagnosis

**Figure 4:**
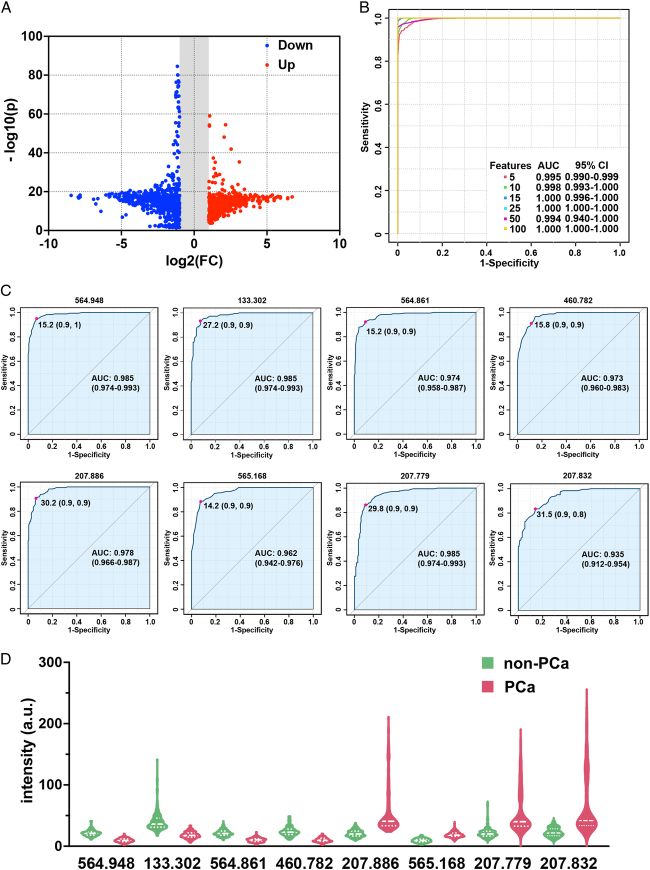
Biomarker analysis and diagnostic model construction. (A) The volcano plot demonstrated the upregulated (red points) and downregulated (blue points) m/z signals in the prostate cancer (PCa) group. The *x*-axes and *y*-axes represent log2 (fold change) and -log10 (*P*-value), respectively. (B) The receiver operative curve of serum metabolic fingerprints for distinguishing PCa subjects from non-PCa subjects based on a different number of selected features. (C) The receiver operative curve curves of eight single m/z for distinguishing PCa subjects from non-PCa subjects. (D) The violin plot demonstrated the differential intensity of eight m/z signals between the PCa subjects (red) and non-PCa subjects (green), and all *P* values were <0.001.

The downregulated and upregulated m/z signals in subjects with PCa are presented in Figure 4A, where the upper left corner displays 642 blue dots and the upper right corner displays 461 red dots, with FC greater than 2 and *P*-value less than 0.05. The receiver operative curve based on various numbers of m/z signals showed an excellent AUC of 100.0% (95% CI: 100.0–100.0%, sensitivity of 100.0%, and specificity of 100.0%) when 100 m/z signals were selected. Moreover, AUC values of 99.5% (95% CI: 99.0–99.9%, sensitivity of 97.8%, and specificity of 95.2%) and 99.8% (95% CI: 99.3–100.0%, sensitivity of 99.4%, and specificity of 97.3%) were achieved when only 5 and 10 m/z signals were selected, respectively (Fig. 4B). Among the m/z signals downregulated in subjects with PCa, m/z 564.948 achieved the best diagnostic performance with an AUC of 98.5% (95% CI: 97.4–99.3%, sensitivity of 95.0%, and specificity of 93.0%), followed by m/z 133.302, 564.861, and 460.782. Among the m/z signals upregulated in the subjects with PCa, m/z 207.886 achieved the best diagnostic performance with an AUC of 97.8% (95% CI: 96.6–98.7%, sensitivity of 90.6%, and specificity of 93.5%), followed by m/z 565.168, 207.779, and 207.832 (Fig. 4C). The relative intensities of the eight m/z signals are shown in Figure 4D (*P*<0.001).

### Construction of a metabolite combination panel and metabolic pathway analysis

**Figure 5:**
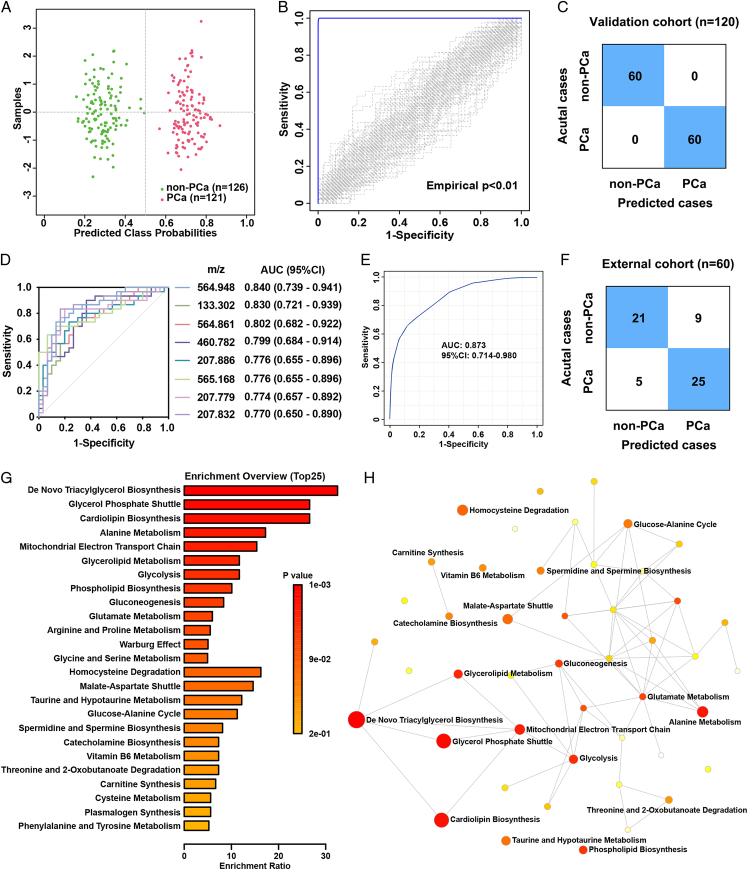
The diagnostic panel and potential pathway analysis. (A) The average of predicted class probabilities of each sample across the 100 cross-validations based on eight selected features. The classification boundary is located at the center (x=0.5, the dotted line). (B) The permutation test of the diagnostic model using eight selected features. (C) The validation cohort (*n*=120) was conducted to evaluate the output model. (D) The receiver operative curve curves of eight single m/z for distinguishing prostate cancer (PCa) subjects from non-PCa subjects in the external cohort. (E) The receiver operative curve for distinguishing PCa subjects from non-PCa subjects based on the combination of eight selected m/z signals in the external cohort. (F) The evaluation of the diagnostic model using eight selected m/z signals in the external cohort (*n*=60). (G) The potential pathway differentially regulated in subjects with PCa and subjects with non-PCa. The enrichment ratio was computed and the color of each bar was correlated to the *P*-value. (H) The potential metabolic network related to the tumorigenesis of PCa.

To further identify serum metabolic biomarkers specific to PCa, we built a diagnostic model based on the eight m/z signals mentioned above. This model accurately divided the studied subjects into two clusters within the training cohort, comprising 121 subjects with PCa and 126 subjects with non-PCa (Fig. 5A). The diagnostic model achieved an enhanced AUC of 100.0% (sensitivity of 100.0%, and specificity of 100.0%) and was validated by a permutation test 100 times, surpassing the analysis of a single m/z signal (Fig. 5B). Subsequently, the output model was applied to the validation cohort, which included 60 subjects with PCa and 60 subjects with non-PCa, resulting in an accurate and precise diagnosis (Fig. 5C). Furthermore, the diagnostic performance of the eight m/z signals was respectively verified in an external cohort, compromising 30 subjects with PCa and 30 subjects with non-PCa (Fig. 5D). The diagnostic model based the combination of eight selected m/z signals achieved an AUC of 87.3% (95% CI: 71.4–98.0%) (Fig. 5E) and approximately divided the subjects into two clusters in the external cohort (Fig. 5F).

Considering statistical parameters such as FC, *P*-value, VIP score, and diagnostic performance, we subsequently identified and filtered 15 m/z signals with significantly different intensities in subjects with PCa compared to those with non-PCa for metabolic pathway analysis. As shown in Figure 5G, in the enrichment analysis based on the Kyoto Encyclopedia of Genes and Genomes (KEGG) database, there were three metabolic pathways potentially related to the tumorigenesis of PCa with an enrichment ratio exceeding 20 and *P*-value less than 0.05, including de novo triacylglycerol biosynthesis, glycerol phosphate shuttle, and cardiolipin biosynthesis. The related networks between the different metabolic pathways are shown in Figure 5H.

### The risk and stage stratification model for PCa based on SMFs by machine learning

**Figure 6:**
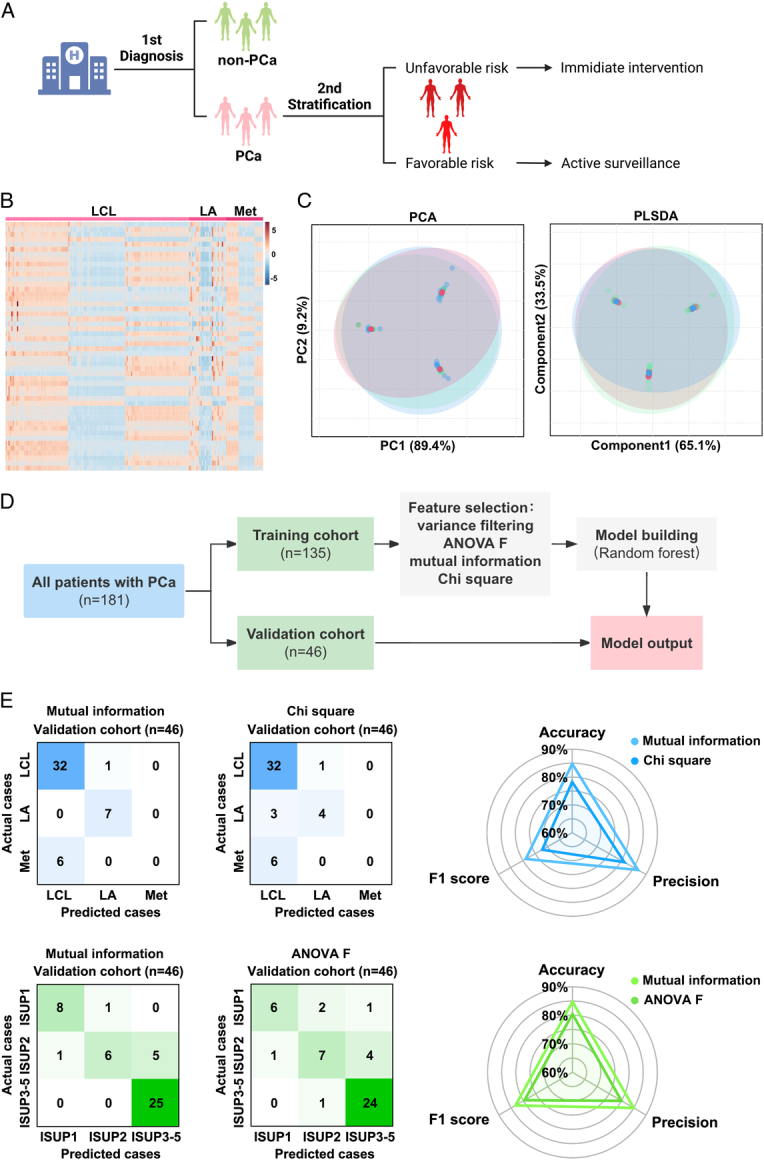
The model based on machine learning for prostate cancer (PCa) risk and stage prediction. (A) The workflow of clinical diagnosis, risk stratification, and treatment allocation of PCa patients. (B) The heatmap of serum metabolic fingerprints for subjects with localized PCa, locally advanced PCa and metastatic PCa. (C) The conventional PCA and PLSDA algorithms for distinguishing the PCa subjects of different clinical stages. (D) The risk and stage stratification of PCa subjects in the training cohort (*n*=135), using different algorithms (variance filtering, ANOVA F, MI, and χ^2^) for feature selection and random forest algorithm for model building. (E) The validation cohort (*n*=46) was conducted to evaluate the output models of stage stratification and risk stratification, including accuracy, precision, and F1 score.

To complement the histopathology-based diagnosis, we determined whether SMFs could be used to guide the risk stratification and stage prediction (Fig. 6A). A heatmap of the SMFs of 181 patients with PCa is shown in Figure 6B. Conventional PCA and PLS-DA algorithms were performed to distinguish subjects with PCa across distinct clinical stages, which failed to divide the serum samples from PCa subjects into three clusters (Fig. 6C). Consequently, we randomly divided all serum samples of the subjects with PCa into a training cohort (*n*=135) and an independent validation cohort (*n*=46) at a ratio of 3:1 for feature selection, model building, and validation (Fig. 6D). For the stage prediction model distinguishing PCa subjects with different clinical stages, the optimized performance with an accuracy of 85.9% was obtained in the training cohort using variance filtering and MI for feature selection and RF for model building. Subsequently, comparable outcomes were obtained in the independent validation cohort, with an accuracy of 84.8%, precision of 86.8%, and F1 score of 78.9%. Another stage prediction model using variance filtering and χ^2^ for feature selection showed optimized performance with an accuracy of 82.2%; similar results were achieved in the independent validation cohort with an accuracy of 78.3%, precision of 81.2%, and F1 score of 72.2% (Fig. 6E). For the risk stratification model distinguishing PCa subjects with different ISUP grades, an optimized performance with an accuracy of 89.6% was obtained in the training cohort using variance filtering and MI for feature selection and RF for model building. Comparable outcomes were observed in the independent validation cohort, with an accuracy of 84.8%, a precision of 85.0%, and an F1 score of 83.3%. Another risk stratification model using variance filtering and ANOVA F for feature selection showed an optimized performance with an accuracy of 82.2%. The efficacy of the model was further confirmed in an independent validation cohort, which yielded comparable results, with an accuracy of 80.4%, precision of 80.0%, and F1 score of 79.6% (Fig. 6E).

## Discussion

To the best of our knowledge, this is the first large population-based prospective study to explore the SMFs of patients with PCa using MALDI-TOF MS analysis. We identified a number of metabolites that differed between the PCa and non-PCa cohorts and successfully built a promising high-performance diagnostic model. We also revealed the potential of SMFs to distinguish low-risk PCa patients from high-risk ones, thereby facilitating precise clinical diagnosis. Finally, we speculated on the underlying metabolic mechanisms of tumorigenesis.

Metabolic reprogramming is one of the hallmarks of malignant tumors, including but not limited to PCa^10,14^. Multiple studies on metabolomics exhibited great potential for metabolic fingerprints for cancer diagnosis and phenotyping^18,19^. As a typical method for generating thousands of peaks, MS can obtain high-throughput metabolomic data, enabling the accurate identification of metabolites^25,27^. Conventional MS analysis is entirely based on the complex pretreatment of large volumes of body fluids or tissue samples, especially gas or liquid chromatography, to enhance the overall analytical performance^40,41^. To facilitate fast detection speed and simplify the procedures, we introduced the MALDI-TOF MS technique using self-designed MoS_2_/C_3_N_4_ as a matrix to obtain an overview of metabolic profiles in only a few seconds without any sample pretreatment. In addition, it only requires microliters of body fluid samples, which enables replicate experiments to lead to more accurate results and conclusions, and is applicable for rare sample analysis. In addition, the tailored matrix employed here showed significant advantages, including a simple synthesis process, adaptation and tolerance to complex body fluid samples, and high-resolution of small metabolite detection. Hence, this approach provides comprehensive SMFs of PCa patients in a fast, economic, and efficient manner, which represents a promising diagnostic tool for large-scale clinical use.

The clinical diagnosis of PCa is based on prostate biopsy, which is performed by physicians according to an elevated serum PSA level, abnormal MRI findings, or palpable prostate nodules. In addition, disease subtyping and staging mainly rely on systematic examinations, such as bone scans and PET-CT. However, these conventional strategies are constrained by high cost, calling for experienced medical professionals, and time consumption, let alone unsatisfactory sensitivity and specificity^42–45^. Recent studies have focused on molecular signatures, including proteins, genomics, and metabolomics, characterizing cancer development and progression^46–49^. Among these, cancer metabolism is partly regulated by signaling and transcriptional networks activated by mutations in oncogenes and tumor suppressors, resulting in metabolic reprogramming^20^. Deciphering small metabolites is a promising way to diagnose cancers and understand the mechanism of tumorigenesis. Although previous studies have demonstrated the potential of metabolic fingerprints in serum and urine for detecting diverse cancers^30–34^, the comprehensive metabolomics of PCa requires additional investigation, and the current metabolic biomarkers are not eminently dependable for clinical application. Compared with those studies dependent on complex deep learning algorithms, our work based on the MALDI-TOF MS technique constructed an excellent diagnostic model based on the conventional OPLS-DA algorithm with an optimal prediction rate of 97.5%. In a PCa-screening scenario, the ability to avoid high false positivity and unnecessary biopsy caused by existing laboratory or imaging tests is desirable and prospective. Meanwhile, we successfully screened out the different m/z signals between the subjects with PCa and those with non-PCa, and in univariate analysis using only one signal m/z signal, the AUC was surprisingly satisfactory. Additionally, we combined a panel of eight significantly specific m/z signals, showing superior performance to a single m/z signal in discriminating PCa from non-PCa, which could dramatically simplify the analytical procedure and facilitate large-scale clinical implementation.

In addition to cancer diagnosis, MALDI-TOF MS can also reveal important clues for potential tumorigenesis mechanisms. According to the enrichment analysis, the m/z signals that were upregulated in subjects with PCa seemed to be involved in the de novo triacylglycerol biosynthesis, glycerol phosphate shuttle, and cardiolipin biosynthesis pathways. Triacylglycerol localized in intracellular lipid droplets is one of the most important attributes for cell stability and prevention of radical damage^50^. As highly dynamic organelles, lipid droplets enable cancer cells to sustain metabolic flexibility through the generation of ATP after beta-oxidation or the deposit of fatty acids to suppress lipotoxicity^51–53^. Glycerol phosphate shuttle, one of the major NADH shuttles, functions as a metabolic hub connecting glycolysis, lipid synthesis, and oxidative phosphorylation, which maintains the energy production to fuel the invasiveness of cancer cells^54,55^. Cardiolipin synthesized exclusively in mitochondria supports oxidative phosphorylation by stabilizing the complexes of the electron transport chain^56^. The accumulation of cardiolipins enhances mitochondrial function to meet the metabolic needs of cancer cells to acquire a survival advantage^57,58^. Nevertheless, additional biological investigations are imperative to elucidate the exact molecular mechanisms that underlie the participation of these metabolites in the tumorigenesis and progression of PCa.

Moreover, the clinical stage and risk stratification of PCa largely determine treatment decisions and prognosis. Current imaging tests for disease staging and subtyping are generally expensive, laborious, and time-consuming^45^. Thus, it is desirable to develop a novel method to refine cancer diagnosis and improve long-term outcomes. Conventional algorithms, including PCA and PLS-DA, failed to classify the subjects into different clinical stages or risk subtypes due to the lack of markedly different features between the cohorts. To overcome this constraint, we used variance filtering to screen out the potential m/z signals and implemented three different methods for feature selection: ANOVA F, MI, and χ^2^. The accuracy of our models based on RF for stage and risk stratification reached 85.9 and 89.6%, respectively, which was verified in the validation cohort. Despite the possibility of sporadic misdiagnoses, this methodology serves as a potent supplementary tool to existing approaches, providing a prompt, economical, and effective means of determining clinical stage and risk stratification.

Our work has several limitations. First, the sample size was restricted and external validation from multiple centers was not performed. To improve the performance and generalizability of this technique, future studies with larger sample sizes and more independent external validation cohorts are required. Second, the use of machine learning algorithms to obtain optimized classifiers for diagnosis may introduce certain limitations. The accuracy and robustness of the diagnostic model can be enhanced by exploring alternative classification algorithms or integrating multiple algorithms. Third, the universal availability of MALDI-TOF MS in all clinical settings may be limited by the requirement for specialized instruments. Therefore, it is imperative to develop user-friendly and portable versions of this technique to facilitate its widespread adoption in routine clinical practice.

## Conclusions

In general, we have established a high-performance SMFs platform based on MALDI-TOF MS and identified PCa-specific metabolites as potential diagnostic markers. Notably, the SMFs analysis only required a blood test, which is promising for universal and large-scale point-of-care applications. Our study identified a potential metabolic pathway involved in PCa tumorigenesis, thereby supporting further biological investigations for precise cancer treatment. Furthermore, our methodology has exhibited potential for improving clinical staging and risk stratification in a timely, cost-effective, and efficient manner. Despite several inherent limitations, this innovative approach represents a significant advancement in PCa research and holds promise for enhancing diagnostic accuracy and guiding clinical decisions. Our work also paves the way for the future development of novel diagnostic tools based on metabolic analysis for diverse malignancies.

## Ethical approval

The present study was approved by the Ethics Committee of Ren Ji Hospital, Shanghai Jiao Tong University School of Medicine (KY2021-030).

## Consent section

Written informed consent was obtained from the patient for publication of this case report and accompanying images. A copy of the written consent is available for review by the Editor-in-Chief of this journal on request.

## Sources of funding

This work was financially supported by the Shanghai Key Clinical Specialty Construction Project Urology (Ren Ji Hospital), the National Natural Science Foundation of China (82272002, 82227801, 81901747, 82203069), Natural Science Foundation of Shanghai, China (22ZR1438500), Program of Shanghai Subject Chief Scientist (19XD1402300), Program for Outstanding Medical Academic Leader (2019LJ11), Shanghai Municipal Health Commission (2020CXJQ03), and National Natural Science Foundation Promotion Project of Ren Ji Hospital, Shanghai Jiao Tong University School of Medicine (RJTJ22-ZD-009, RJTJ22-MS-012).

## Author contribution

X.F.: conceptualized, investigated, supervised, wrote, and edited the work; X.D.: investigated and performed the formal analysis; J.W.: validated the work and worked on the data curation; J.L.: performed sample collection and worked on project administration; Y.G.: performed the visualization; Z.Z.: worked on the software; Y.Z.: validated and edited the work; B.D. and L.D.: reviewed and edited the work; J.P.: supervised the work and provided resources; Z.C. and Q.F.: provided resources; W.S.: supervised and reviewed the work, and worked on the methodology; S.X.: conceptualized, supervised, reviewed, and edited the work; W.X.: conceptualized, supervised, and reviewed the work, and provided resources. All authors joined in the critical discussion and edited the work.

## Conflicts of interest disclosure

The authors declare no potential conflicts of interest.

## Research registration unique identifying number (UIN)

1. The name of the registry: Research Registry.
2. The study was registered at https://www.researchregistry.com/
3. Registration ID: researchregistry9864

## Guarantor

Xiaochen Fei, Shaowei Xie, and Wei Xue.

## Data availability statement

The data that support the findings of this study are available from the corresponding author, Shaowei Xie, upon reasonable request.

## Provenance and peer review

Not commissioned, externally peer-reviewed.

## Supplementary Material

SUPPLEMENTARY MATERIAL
