# Supplementary material for: Precise diagnosis and risk stratification of prostate cancer by comprehensive serum metabolic fingerprints: a prediction model study
Source: Int J Surg. 2024 Jan 4;110(3):1450–62. doi: 10.1097/JS9.0000000000001033 (PMC10942223; doi:10.1097/JS9.0000000000001033)
Supplement: SUPPLEMENTARY MATERIAL [file js9-110-1450-s001.docx]

| Section/topic | Item | Development or validation? | Checklist item | Page | |
| --- | --- | --- | --- | --- | --- |
| Title and abstract | | | | |  |
| Title | 1 | D;V | Identify the study as developing and/or validating a multivariable prediction model, the target population, and the outcome to be predicted | 1 | |
| Abstract | 2 | D;V | Provide a summary of objectives, study design, setting, participants, sample size, predictors, outcome, statistical analysis, results, and conclusions | 1 | |
| Introduction | | | | |  |
| Background and objectives | 3a | D;V | Explain the medical context (including whether diagnostic or prognostic) and rationale for developing or validating the multivariable prediction model, including references to existing models | 2 | |
|  | 3b | D;V | Specify the objectives, including whether the study describes the development or validation of the model, or both | 2 | |
| Methods | | | | |  |
| Source of data | 4a | D;V | Describe the study design or source of data (for example, randomised trial, cohort, or registry data), separately for the development and validation data sets, if applicable | 2-3 | |
|  | 4b | D;V | Specify the key study dates, including start of accrual; end of accrual; and, if applicable, end of follow-up | 2-3 | |
| Participants | 5a | D;V | Specify key elements of the study setting (for example, primary care, secondary care, general population) including number and location of centres | 2-3 | |
|  | 5b | D;V | Describe eligibility criteria for participants | 2-3 | |
|  | 5c | D;V | Give details of treatments received, if relevant | NA | |
| Outcome | 6a | D;V | Clearly define the outcome that is predicted by the prediction model, including how and when assessed | 3 | |
|  | 6b | D;V | Report any actions to blind assessment of the outcome to be predicted | NA | |
| Predictors | 7a | D;V | Clearly define all predictors used in developing the multivariable prediction model, including how and when they were measured | 3-4 | |
|  | 7b | D;V | Report any actions to blind assessment of predictors for the outcome and other predictors | NA | |
| Sample size | 8 | D;V | Explain how the study size was arrived at. | NA | |
| Missing data | 9 | D;V | Describe how missing data were handled (for example, complete-case analysis, single imputation, multiple imputation) with details of any imputation method | NA | |
| Statistical analysis methods | 10a | D | Describe how predictors were handled in the analyses | 4 | |
|  | 10b | D | Specify type of model, all model-building procedures (including any predictor selection), and method for internal validation | 4 | |
|  | 10c | V | For validation, describe how the predictions were calculated | NA | |
|  | 10d | D;V | Specify all measures used to assess model performance and, if relevant, to compare multiple models | 4 | |
|  | 10e | V | Describe any model updating (for example, recalibration) arising from the validation, if done | NA | |
| Risk groups | 11 | D;V | Provide details on how risk groups were created, if done | NA | |
| Development *v* validation | 12 | V | For validation, identify any differences from the development data in setting, eligibility criteria, outcome, and predictors | NA | |
| Results | | | | |  |
| Participants | 13a | D;V | Describe the flow of participants through the study, including the number of participants with and without the outcome and, if applicable, a summary of the follow-up time. A diagram may be helpful | 5 | |
|  | 13b | D;V | Describe the characteristics of the participants (basic demographics, clinical features, available predictors), including the number of participants with missing data for predictors and outcome | 5 | |
|  | 13c | V | For validation, show a comparison with the development data of the distribution of important variables (demographics, predictors and outcome). | NA | |
| Model development | 14a | D | Specify the number of participants and outcome events in each analysis | 5-6 | |
|  | 14b | D | If done, report the unadjusted association between each candidate predictor and outcome | NA | |
| Model specification | 15a | D | Present the full prediction model to allow predictions for individuals (that is, all regression coefficients, and model intercept or baseline survival at a given time point) | 5-6 | |
|  | 15b | D | Explain how to use the prediction model | 5-6 | |
| Model performance | 16 | D;V | Report performance measures (with CIs) for the prediction model | 5-6 | |
| Model updating | 17 | V | If done, report the results from any model updating (that is, model specification, model performance) | NA | |
| Discussion | | | | |  |
| Limitations | 18 | D;V | Discuss any limitations of the study (such as nonrepresentative sample, few events per predictor, missing data) | 7-8 | |
| Interpretation | 19a | V | For validation, discuss the results with reference to performance in the development data, and any other validation data | NA | |
|  | 19b | D;V | Give an overall interpretation of the results, considering objectives, limitations, results from similar studies, and other relevant evidence | 6-8 | |
| Implications | 20 | D;V | Discuss the potential clinical use of the model and implications for future research | 6-8 | |
| Other information | | | | |  |
| Supplementary information | 21 | D;V | Provide information about the availability of supplementary resources, such as study protocol, Web calculator, and data sets | NA | |
| Funding | 22 | D;V | Give the source of funding and the role of the funders for the present study | NA | |
