# Supplementary material for: Precise diagnosis and risk stratification of prostate cancer by comprehensive serum metabolic fingerprints: a prediction model study
Source: Int J Surg. 2024 Jan 4;110(3):1450–62. doi: 10.1097/JS9.0000000000001033 (PMC10942223; doi:10.1097/JS9.0000000000001033)
Supplement: SUPPLEMENTARY MATERIAL [file js9-110-1450-s002.docx]

**Precise Diagnosis and Risk Stratification of Prostate Cancer by Comprehensive Serum Metabolic Fingerprints: A Prospective Pilot Study**


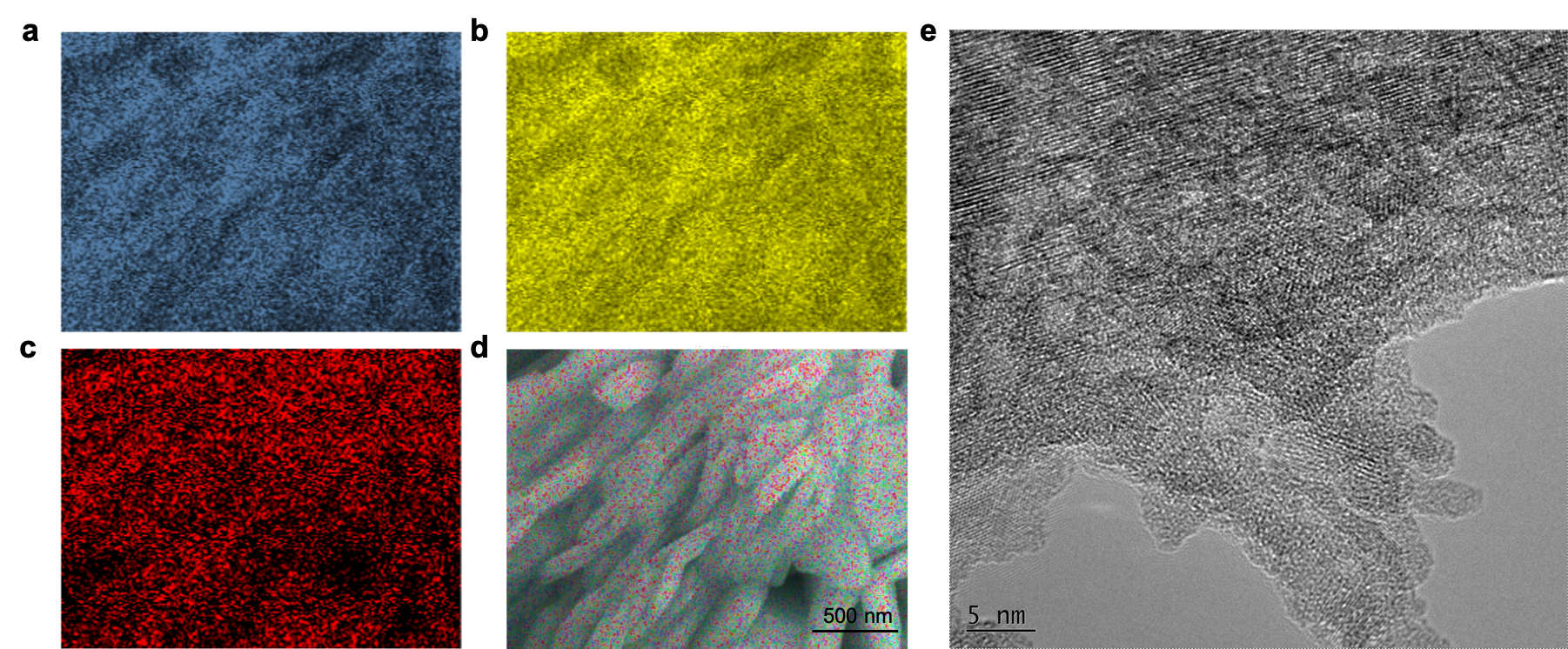


**Fig. S1** The elemental mapping analysis and the high-resolution TEM of MoS_2_/C_3_N_4_. The elements including S (a), Mo (b) and C (d) were detected and the emerged mapping evidenced the successful synthesis of MoS_2_/C_3_N_4_. (e) The high-resolution TEM of MoS_2_/C_3_N_4_.


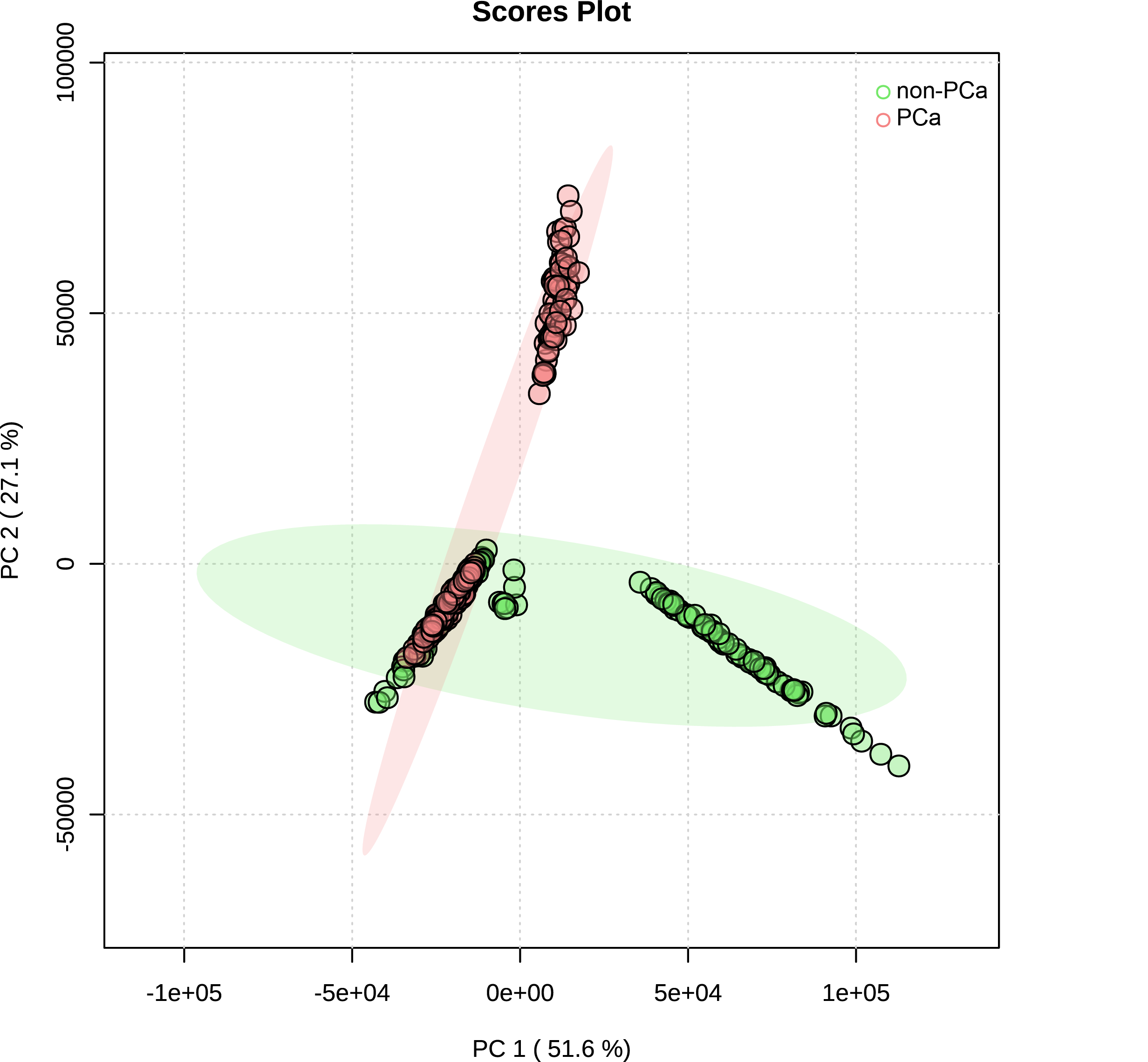


**Fig. S2** The PCA score plot. Classification for the subjects with PCa (red points) and the subjects with non-PCa (green points).
