# Supplementary material for: Precise diagnosis and risk stratification of prostate cancer by comprehensive serum metabolic fingerprints: a prediction model study
Source: Int J Surg. 2024 Jan 4;110(3):1450–62. doi: 10.1097/JS9.0000000000001033 (PMC10942223; doi:10.1097/JS9.0000000000001033)
Supplement: SUPPLEMENTARY MATERIAL [file js9-110-1450-s003.docx]

|  | **PCa (n=181)** | **Non-PCa (n=186)** |
| --- | --- | --- |
| **Median age (IQR)** | 71.0 (67.0-75.0) | 67.0 (63.0-70.0) |
| **No. PSA (%)** |  | |
| <4 ng/ml | 1 (0.6) | 8 (4.3) |
| 4-10 ng/ml | 61 (33.7) | 111 (59.7) |
| 10-20 ng/ml | 45 (24.9) | 45 (24.2) |
| ≥20 ng/ml | 74 (40.9) | 22 (11.8) |
| **No. Gleason score (%)** |  | NA |
| ISUP 1 | 36 (19.9) |  |
| ISUP 2 | 47 (26.0) |  |
| ISUP 3 | 52 (28.7) |  |
| ISUP 4 | 35 (19.3) |  |
| ISUP 5 | 11 (6.1) |  |
| **No. Clinical stage (%)** |  | NA |
| Localised | 129 (71.3) |  |
| Locally-advanced | 26 (14.4) |  |
| Metastatic | 26 (14.4) |  |
